# Supplementary material for: Effect of a Novel Food Rich in Miraculin on the Oral Microbiome of Malnourished Oncologic Patients with Dysgeusia
Source: Cancers (Basel). 2024 Oct 8;16(19):3414. doi: 10.3390/cancers16193414 (PMC11475728; doi:10.3390/cancers16193414)
Supplement: Supplementary file 1 [file cancers-16-03414-s001.zip › cancers-3208070-supplementary.pdf]

cancers MDPI

Table S1. Nutritional composition of the food supplement enriched in miraculin (DMB) and placebo.

		Standard Dose of DMB (150 mg DMB + 150 mg Strawberry Freeze-Dried)	High Dose of DMB (300 mg DMB)	Placebo (300 mg Strawberry Freeze-Dried)
Energy	kcal	0.99	1	0.97
Carbohydrates	mg	194	234	154
Sugars	mg	156	162	150
Fiber	mg	26	6	46
Proteins	mg	20	15	24
Lipids	mg	9	5	12
Saturated fatty acids	mg	2	2	1
Sodium chloride	mg	0.1	0.1	0.03
Humidity	mg	4	4	5
Ash	mg	12	14	15
Miraculin	mg	2,8	5,6	0

Nutritional composition provided by Medicinal Gardens, S.L.

Table S2. Cancer types and chemotherapy characteristics of the general population.

		Standard Dose of DMB	High Dose of DMB	Placebo	*p*-Value
Type of cancer					
Head and neck	%	0.00	9.10	0.00	
Colorectal	%	30.00	27.30	20.00	
Esophagus	%	10.00	0.00	10.00	
Stomach	%	0.00	9.10	10.00	
Liver	%	0.00	9.10	10.00	
Breast	%	10.00	18.20	10.00	0.895
Neuroendocrine	%	10.00	0.00	0.00	
Ovary	%	10.00	18.20	0.00	
Pancreas	%	10.00	9.10	10.00	
Lung	%	10.00	0.00	10.00	
Others	%	10.00	0.00	20.00	
Chemotherapy	%	100.0	100.0	100.0	1
Radiotherapy	%	20.00	12.50	0.00	0.594
Smoking status (yes)	%	30.00	27.30	40.00	0.605
Alcohol use (yes)	%	20.00	27.30	10.00	0.393
Dental disease (yes)	%	0.00	0.00	0.00	1

Table S3. Distribution of phyla and alpha diversity indices for microbiota of saliva from cancer patients of the CLINMIR study.

Phylum	Standard Dose DMB (150 mg) (n=8)			High Dose DMB (300 mg) (n=6)			Placebo (n=7)			*p*-Value		
	Baseline	1 month	3 months	Baseline	1 month	3 months	Baseline	1 month	3 months	Treatment (T)	Time (t)	T x t
Actinobacteriota	0.6 (0.2 - 2.4)	0.5 (0.09 - 2.0)	0.5 (0.2 - 2.6)	0.7 (0.2 - 0.9)	0.5 (0.4 0.8)	0.4 (0.2 - 0.5)	0.6 (0.2 - 1.4)	0.7 (0.2 - 2.3)	0.6 (0.2 - 1.6)	0.469	0.556	0.926
Bacteroidota	0.01 (0.005 - 0.02)	0.01 (0.007 - 0.02)	0.03 (0.01 - 0.05)	0.01 (0.005 - 0.03)	0.01 (0.009 - 0.06)	0.01 (0.01 - 0.01)	0.01 (0.004 - 0.02)	0.01 (0.007 - 0.06)	0.01 (0.005 - 0.01)	0.815	0.119	0.180
Bacillota	99.2 (97.0 - 99.6)	99.1 (97.6 - 99.7)	99.2 (96.6 - 99.7)	99 (98.2 - 99.8)	99.3 (98.7 - 100)	99.5 (99 - 99.6)	98.9 (98.1 - 99.7)	98.9 (95.1 - 99.3)	98.9 (98.3 - 99.4)	0.254	0.630	0.620

Fusobacteria	0.02 (0.004 - 0.03)	0.01 (0.004 - 0.05)	0.03 (0.004 - 0.06)	0.01 (0.01 - 0.01)	0.01 (0.01 - 0.01)	0.01 (0.006 - 0.05)	0.006 (0.004 - 0.04)	0.01 (0.003 - 0.04)	0.007 (0.007 - 0.007)	0.521	0.619	0.905
Pseudomonadota	0.1 (0.05 - 0.3)	0.1 (0.06 - 0.4)	0.12 (0.05 - 0.5)	0.06 (0.03 - 0.3)	0.07 (0.03 - 0.2)	0.07 (0.04 - 0.4)	0.1 (0.06 - 0.2)	0.2 (0.08 - 0.4)	0.2 (0.08 - 0.3)	0.534	0.343	0.187
Saccharibacteria	0.04 (0.01 - 0.4)	0.08 (0.01 - 0.3)	0.05 (0.008 - 0.4)	0.05 (0.02 - 0.3)	0.07 (0.05 - 0.3)	0.06 (0.01 - 0.1)	0.2 (0.004 - 0.4)	0.1 (0.03 - 2)	0.1 (0.05 - 0.3)	0.252	0.310	0.391
Shannon index	0.5 (0.2 - 1.6)	0.6 (0.2 - 1.3)	0.5 (0.2 - 1.7)	0.6 (0.2 - 1.1)	0.5 (0.3 - 0.9)	0.4 (0.2 - 0.7)	0.7 (0.3 - 1.1)	0.7 (0.5 - 2.5)	0.7 (0.4 - 1.0)	0.686	0.878	0.950
Simpson's index	0.2 (0.1 - 0.6)	0.2 (0.1 - 0.5)	0.2 (0.1 - 0.7)	0.2 (0.1 - 0.3)	0.1 (0.1 - 0.3)	0.1 (0.1 - 0.2)	0.2 (0.1 - 0.4)	0.2 (0.1 - 0.9)	0.2 (0.1 - 0.3)	0.711	0.926	0.908
Chao1 index	36.7 (22.3 - 68.3)	37.8 (25.1 - 75.0)	36.8 (20.5 - 57.5)	36.4 (21.5 - 49.3)	42.2 (34.0 - 85.0)	32.5 (23.6 - 45.5)	37.0 (29.0 - 55.0)	46.8 (25.0 - 71.5)	38.2 (28.5 - 56.0)	0.772	0.656	0.740
Beta diversity	0.08 (0.02 – 0.1)	0.07 (0.03 – 0.3)	0.1 (0.004 – 0.6)	0.2 (0.003 – 1.0)	0.1 (0.1 – 0.2)	0.05 (0.01 – 0.7)	0.1 (0.03 – 0.6)	0.1 (0.08 – 0.6)	0.05 (0.03 – 0.5)	0.140	0.299	0.213

Values are presented as median and range. General linear mixed models (GLM) of variance (ANOVA) were used to evaluate differences between means for treatment, time, and treatment x time.

Table S4. Distribution of selected families for microbiota of saliva from cancer patients of the CLINMIR study.

	**Standard Dose DMB (150 mg) (n=8)**			**High Dose DMB (300 mg) (n=6)**			**Placebo (n=7)**			***p*-Value**		
Selected families	Baseline	1 month	3 months	Baseline	1 month	3 months	Baseline	1 month	3 months	Treatment (T)	Time (t)	T x t
Streptococcaceae	69.4 (61.3 - 71.2)	64.3 (56.5 - 75.2)	68.7 (37.1 - 77.2)	72.0 (53.0 - 94.0)	74.4 (60.0 - 84.5)	69.0 (53.0 - 84.9)	64.9 (49.6 - 85.2)	63.0 (33.6 - 76.8)	68.0 (41.2 - 72.5)	0.333	0.545	0.586
Veillonellaceae	10.5 (3.6 - 20.9)	6.9 (3.5 - 27.8)	3.9 (0.07 - 6.0)	5.3 (1.7 - 8.1)	6.8 (4.6 - 15.8)	7.4 (3.5 - 15.5)	11.0 (3.1 - 24.4)	6.5 (2.9 - 14.0)	5.9 (4.6 - 8.4)	0.436	0.856	0.906
Carnobacteriaceae	4.9 (1.7 - 14.7)	3.7 (2.2 - 8.0)	4.9 (1.4 - 9.2)	1.6 (0.09 - 7.4)	1.6 (0.3 - 4.8)	4.0 (0.8 - 23.3)	3.0 (1.4 - 8.8)	4.8 (1.3 - 7.4)	2.7 (1.3 - 3.2)	0.733	0.463	0.936
Lachnospiraceae	3.8 (1.5 - 5.2)	5.4 (1.2 - 10.2)	4.2 (1.4 - 27.0)	1.5 (0.08 - 17.9)	4.8 (0.5 - 6.9)	2.5 (0.2 - 18.9)	3.6 (2.4 - 8.4)	8.2 (1.7 - 15.9)	4.9 (0.9 - 15.4)	0.128	0.312	0.345
Aerococcaceae	3.5 (0.2 - 7.9)	3.1 (0.4 - 14.3)	0.8 (0.03 - 15.0)	1.4 (0.3 - 8.5)	2.0 (1.3 - 6.8)	1.3 (0.2 - 4.8)	2.0 (0.1 - 8.4)	2.4 (0.2 - 11.9)	0.6 (0.2 - 14.3)	0.793	0.734	0.870
Lactobacillaceae	0.9 (0.1 - 13.5)	1.3 (0.1 - 17.7)	3.7 (0.2 - 12.9)	2.3 (0.7 - 7.0)	0.5 (0.4 - 8.7)	2.5 (0.3 - 11.9)	2.4 (0.08 - 12.9)	2.3 (0.1 - 13.3)	0.4 (0.1 - 9.0)	0.124	0.312	0.162
Bacillaceae	0.9 (0.4 - 2.1)	0.7 (0.5 - 1.3)	0.9 (0.6 - 1.6)	0.3 (0.1- 1.2)	0.4 (0.2 - 0.6)	0.7 (0.3 - 3.2)	0.6 (0.3 - 1.4)	0.8 (0.4 - 0.9)	0.5 (0.3 - 0.6)	0.478	0.395	0.764
Eubacteriales Family XIII. Incertae Sedis	0.6 (0.07 - 1.5)	0.5 (0.08 - 2.5)	0.7 (0.2 - 6.8)	0.5 (0.05 - 1.1)	0.4 (0.2 - 0.9)	0.4 (0.1 - 0.7)	1.0 (0.2 - 3.6)	0.7 (0.06 - 5.9)	0.8 (0.03 - 8.7)	0.405	0.373	0.682
Enterococcaceae	0.5 (0.2 - 1.4)	0.4 (0.3 - 0.8)	0.5 (0.4 - 0.9)	2.6 (0.03 - 12.1)	0.1 (0.04 - 5.8)	0.4 (0.08 - 2.4)	0.3 (0.1 - 0.8)	0.5 (0.2 - 0.6)	0.3 (0.2 - 11.4)	0.187	0.489	0.174
Listeriaceae	0.5	0.3	0.6	0.09	0.2	0.3	0.2	0.3	0.2	0.445	0.373	0.623

	(0.1 - 1.1)	(0.2 - 0.6)	(0.3 - 0.8)	(0.01 - 0.7)	(0.06 - 0.3)	(0.08 - 1.8)	(0.08 - 0.7)	(0.1 - 0.4)	(0.07 - 0.2)			
Actinomycetaceae	0.3 (0.1 - 1.2)	0.4 (0.05 - 1.1)	0.2 (0.05 - 0.6)	0.2 (0.08 - 0.5)	0.3 (0.07 - 0.4)	0.2 (0.1 - 0.3)	0.4 (0.2 - 1.3)	0.4 (0.1 - 1.3)	0.3 (0.1 - 0.7)	0.961	0.413	0.481
Micrococcaceae	0.2 (0.01 - 0.3)	0.06 (0.01 - 0.3)	0.09 (0.04 - 0.3)	0.07 (0.02 - 0.5)	0.05 (0.02 - 0.1)	0.03 (0.009 - 0.1)	0.09 (0.02 - 0.3)	0.2 (0.02 - 0.3)	0.07 (0.01 - 0.7)	0.110	0.286	0.161
Staphylococcaceae	0.2 (0.1 - 0.5)	0.2 (0.1 - 0.4)	0.3 (0.2 - 0.4)	0.1 (0.03 - 0.5)	0.1 (0.07 - 0.2)	0.3 (0.06 - 0.9)	0.2 (0.07 - 0.4)	0.3 (0.2 - 1.6)	0.1 (0.1 - 0.3)	0.629	0.734	0.821
Clostridiaceae	0.1 (0.04 - 0.2)	0.1 (0.03 - 0.4)	0.2 (0.03 - 0.5)	0.08 (0.02 - 0.4)	0.1 (0.04 - 0.3)	0.08 (0.02 - 0.5)	0.2 (0.04 - 0.7)	0.2 (0.03 - 1.2)	0.2 (0.02 - 1.4)	0.508	0.338	0.847
Shannon index	1.2 (1.1 - 1.4)	1.3 (1.1 - 1.7)	1.3 (1.0 - 1.9)	1.1 (0.3 - 1.7)	1.1 (0.7 - 1.5)	1.2 (0.7 - 1.6)	1.4 (0.7 - 1.6)	1.4 (1.0 - 2.2)	1.3 (1.0 - 1.9)	0.668	0.536	0.767
Simpson's index	0.5 (0.5 - 0.6)	0.6 (0.4 - 0.7)	0.5 (0.4 - 0.8)	0.5 (0.1 - 0.7)	0.4 (0.3 - 0.6)	0.5 (0.3 - 0.7)	0.6 (0.3 - 0.7)	0.6 (0.4 - 0.8)	0.5 (0.5 - 0.8)	0.726	0.493	0.797
Chao1 index	42.3 (28.0 - 145.0)	32.3 (27.3 - 75.0)	37.2 (26.7 - 65.9)	44.6 (29.0 - 66.5)	32.7 (24.2 - 40.6)	34.9 (31.0 - 52.0)	39.6 (20.7 - 82.0)	36.5 (25.1 - 46.0)	42.1 (19.7 - 62.5)	0.406	0.251	0.495
Beta diversity	0.2 (0.09 – 0.2)	0.2 (0.08 – 0.3)	0.2 (0.08 – 0.7)	0.3 (0.1 – 1.0)	0.2 (0.1 – 0.3)	0.2 (0.1 – 0.7)	0.3 (0.1 – 0.6)	0.2 (0.2 – 0.6)	0.2 (0.1 – 0.7)	0.204	0.459	0.398

Values are presented as median and range. General linear mixed models (GLM) of variance (ANOVA) were used to evaluate differences between means for treatment, time, and treatment x time.
